# Degummed crude canola oil, sire breed and gender effects on intramuscular long-chain omega-3 fatty acid properties of raw and cooked lamb meat

**DOI:** 10.1186/s40781-017-0143-7

**Published:** 2017-08-21

**Authors:** Aaron Ross Flakemore, Bunmi Sherifat Malau-Aduli, Peter David Nichols, Aduli Enoch Othniel Malau-Aduli

**Affiliations:** 1 Animal Science and Genetics, Tasmanian Institute of Agriculture, School of Land and Food, Faculty of Science, Engineering and Technology, University of Tasmania, Private Bag 54 Sandy Bay, Hobart, TAS 7001 Australia; 2 College of Medicine and Dentistry, Division of Tropical Health and Medicine, James Cook University, Townsville, QLD 4811 Australia; 3 Commonwealth Scientific and Industrial Research Organisation, Food, Nutrition and Bi-based Products, Oceans and Atmosphere, G.P.O. Box 1538, Hobart, TAS 7001 Australia; 4 Animal Genetics and Nutrition, Veterinary Sciences, College of Public Health, Medical and Veterinary Sciences, Division of Tropical Health and Medicine, James Cook University, Townsville, QLD 4811 Australia

**Keywords:** Canola oil, Lamb, Cooking, Omega-3 fatty acids, Meat quality

## Abstract

**Background:**

Omega-3 long-chain (≥C_20_) polyunsaturated fatty acids (ω3 LC-PUFA) confer important attributes to health-conscious meat consumers due to the significant role they play in brain development, prevention of coronary heart disease, obesity and hypertension. In this study, the ω3 LC-PUFA content of raw and cooked *Longissimus thoracis et lumborum* (LTL) muscle from genetically divergent Australian prime lambs supplemented with dietary degummed crude canola oil (DCCO) was evaluated.

**Methods:**

Samples of LTL muscle were sourced from 24 first cross ewe and wether lambs sired by Dorset, White Suffolk and Merino rams joined to Merino dams that were assigned to supplemental regimes of degummed crude canola oil (DCCO): a control diet at 0 mL/kg DM of DCCO (DCCOC); 25 mL/kg DM of DCCO (DCCOM) and 50 mL/kg DCCO (DCCOH). Lambs were individually housed and offered 1 kg/day/head for 42 days before being slaughtered. Samples for cooked analysis were prepared to a core temperature of 70 °C using conductive dry-heat.

**Results:**

Within raw meats: DCCOH supplemented lambs had significantly (*P* < 0.05) higher concentrations of eicosapentaenoic (EPA, 20:5ω3) and EPA + docosahexaenoic (DHA, 22:6ω3) acids than those supplemented with DCCOM or DCCOC; Dorset sired lambs contained significantly (*P* < 0.05) more EPA and EPA + DHA than other sire breeds; diet and sire breed interactions were significant (*P* < 0.05) in affecting EPA and EPA + DHA concentrations. In cooked meat, ω3 LC-PUFA concentrations in DCCOM (32 mg/100 g), DCCOH (38 mg/100 g), Dorset (36 mg/100 g), White Suffolk (32 mg/100 g), ewes (32 mg/100 g) and wethers (33 mg/100 g), all exceeded the minimum content of 30 mg/100 g of edible cooked portion of EPA + DHA for Australian defined ‘source’ level ω3 LC-PUFA classification.

**Conclusion:**

These results present that combinations of dietary degummed crude canola oil, sheep genetics and culinary preparation method can be used as effective management tools to deliver nutritionally improved ω3 LC-PUFA lamb to meat consumers.

## Background

Ruminant meats are important components of the human diet, particularly in Western countries [1, 2]. However, perceived negative health effects associated with increased saturated fatty acid intakes (i.e. cardiovascular disease, cancers and obesity) are now embedded in the public consciousness [3–5]. Subsequently, consumers are favouring alternative healthier foods, or leaner meats containing less saturated fat and higher concentrations of health promoting omega-3 polyunsaturated fatty acids [6, 7]. Therefore, an improvement in the content of health beneficial omega-3 long-chain (≥C_20_) polyunsaturated fatty acids (ω3 LC-PUFA); eicosapentaenoic (EPA, 20:5ω3) and docosahexaenoic (DHA, 22:6ω3) in lamb has become a high priority for the Australian meat sector [8–10].

Nutritional intervention through dietary lipid supplementation has emerged as an area of interest to manipulate intramuscular fat (IMF) properties of red meats with targeted flow on beneficial effects to human health [11, 12]. However, endeavours to successfully meet consumer demands for healthier meat products via dietary lipid supplementation have been shown to be complex due to the intricate interactions between dietary factors and rumen metabolism; namely, the level and source of dietary lipids [13, 14], the basal diet [13, 14] and the potentially limited capacity of ω3 LC-PUFA to escape rumen biohydrogenation for subsequent metabolic distribution and partitioning into muscle and other tissues [11, 15–17].

In addition to diet, lamb genetics can dictate the nature of the nutritional status of ω3 LC-PUFA content in meat [18–20], and the response of different sheep breeds to nutritional supplementation. Moreover, genetic predispositions between sheep types based on production traits (i.e. wool/meat/milk) through extensive breeding and crossbreeding directs that fatty acid properties of sheep may respond differently to changes in diet [20, 21]. Therefore, interaction responses between supplementary nutritional input through omega-3 oil sources and sheep types developed for specific production purposes and subsequent effects on meat lipid properties require ongoing investigations are needed.

Increases in commercial canola production throughout Australia have now made canola oil easily available and at a competitively affordable price [22, 23]. To our current knowledge, however, there is a dearth in published data assessing the optimum level of inclusion of degummed canola oil in Australian prime lamb diets for enhancing the health beneficial LC-omega-3 fatty acid concentrations in meats for consumers. In addition, despite a number of studies quantifying the effect of cooking on lamb meat fatty acid properties [24–27], there is very little published information emanating from sheep under on-farm management conditions, assessing the impacts of dietary supplementation, breed, gender and their interactions on FA composition within the raw and cooked meat as prepared for consumption. The objective of this study was to examine the effects of dietary degummed crude canola oil supplementation and its interactions amongst genetically divergent Australian sheep types on selected nutrients (moisture and intramuscular fat content), fatty acid composition and content (with particular emphasis on ω-3 LC-PUFA), and their retention properties in raw and cooked meats. The hypotheses for this study were: *(i) DCCO can be successfully included in sheep rations to beneficially manipulate fatty acids in raw meat, which will transpire to the final cooked product, consequently generating a nutritionally beneficial product to the consumer; (ii) genetically divergent lambs demonstrate differences in meat lipid properties that can be amended through dietary DCCO intervention; and (iii) gender plays a minor role in the fatty acid composition of prime lambs supplemented with canola oil.*

## Methods

### Animals, management and sample collection

The experimental conditions for this study have been reported previously [28–30]. To reiterate, the feeding phase of this experiment comprised twenty-four (24) individually housed, 6-month-old, first cross weaner lambs that were assigned to one of three dietary degummed crude canola oil (DCCO) treatments: 1) a control diet (DCCOC) at 0 mL/kg DM of DCCO; 2) an intermediate diet (DCCOM) at a volume 25 mL/kg DM of DCCO; and 3) a high rate (DCCOH) of added DCCO at 50 mL/kg DM. The dietary treatments were based on two wheat/barley-based rations, one concentrate without DCCO, and the other with canola replacing the barley component in the concentrates at an oil inclusion of 5%. A full account of the ingredient profiles, proximate compositions and fatty acid profiles is given in Table 1. Each dietary group comprised of first-cross ewe and wether progeny from Merino dams joined to Dorset, White Suffolk, and Merino sires at a mating ratio of 1:100. Following an initial 21-day adjustment period, each animal was provided 1 kg of concentrate daily for 42 days. Ad libitum*,* access to lucerne hay and water was provided throughout the experimental period. At the completion of the feeding trial, all experimental animals, except four (4) purebred Merino ewes, were slaughtered and processed as per Australian commercial operating procedures, thus bringing the number of experimental animals for analyses to 20. Samples of commercially prepared loin chop portions (one each for raw and cooked analysis) comprising the *longissimus thoracis et lumborum (LTL)* muscle were excised from each carcass, and stored at −20 °C until required for analysis.

**Table 1:** Ingredients and chemical compositions of experimental diets

	Concentrate 1 (Added DCCO)	Concentrate 2 (no added DCCO)	Lucerne hay
*Ingredients %*			
Barley	-	25.87	-
Wheat 21%	17.00	25.00	-
Mill mix	21.18	20.17	-
Paddy rice	28.00	7.26	-
Lupins	-	16.00	-
Canola meal	15.44	-	-
Canola oil	5.00	-	-
GOMF	8.00	-	-
Limestone 37%	1.774	2.09	-
Ammonium sulphate	1.25	1.25	-
Salt	1.00	1.00	-
Acid buff	0.625	0.625	-
Sodium bicarb	0.625	0.625	-
Sheep premix	0.10	0.10	-
Bovatec 20%	0.01	0.01	-
*Proximate composition* ^a^			
DM	91.8	90.9	85.6
CP	12.7	10.4	17.0
ADF	8.0	9.0	44.9
NDF	20.0	21.1	55.2
EE	6.2	2.1	1.5
Ash	9.7	8.9	6.8
TDN	75.7	72.0	55.4
ME (MJ/kg)	12.1	11.4	8.4
*Fatty acid (% TFA)* ^b^			
16:0	26.1	32.1	-
18:0	3.8	3.8	-
18:1ω9	41.9	16.5	-
18:2ω6	6.9	17.7	-
18:3ω3	0.5	1.6	-
∑SFA	38.6	41.2	-
∑MUFA	48.7	23.3	-
∑PUFA	35.0	12.6	-
∑ω6	20.1	10.2	-
∑ω3	14.9	0.9	-

^a^%DM (percentage dry matter), CP (crude protein), ADF (acid detergent fibre), NDF (neutral detergent fiber), EE (ether extractable fat), TDN (total digestible nutrients), ME (metabolisable energy). ^b^ ∑SFA is the sum total of 14:0, 15:0, 16:0, 17:0, 18:0, i15:0, a15:0, i16:0, 16:0FALD, i17:0, i18:0, 18:0FALD, 20:0, 22:0, 23:0, 24:0. ∑MUFA is the sum total of 16:1ω7c, 18:1ω9c, 18:1ω7c, 18:1ω7t, 14:1, 16:1ω9c, 16:1ω7t, 16:1ω5c, 16:1ω13t, 17:1ω8c + a17:0, 17:1, 18:1a, 18:1b, 18:1c, 19:1a, 19:1b, 18:1FALD, 20:1ω11c, 20:1ω9c, 20:1ω7c, 20:1ω5c, 22:1ω11c, 22:1ω9c, 24:1ω9c. ∑PUFA is the sum total of 18:2ω6, 18:3ω3, 20:4ω6, 20:5ω3, 22:6ω3, 22:5ω3, 18:3ω6, 18:4ω3, 18:2ω6CLAa, 18:2ω6CLAb, 18:2ω6CLAc, 20:3, 20:3ω6, 20:4ω3, 20:2ω6, 22:5ω6, 22:4ω6. ∑ω6 = is the combined sum total of 18:2ω6, 20:4ω6, 18:3ω6, 20:3ω6, 20:2ω6, 22:5ω6, 22:4ω6. ∑ω3 = is the combined sum total of 18:3ω3, 20:5ω3, 22:6ω3, 22:5ω3, 18:4ω3, 20:4ω3. Proximate analysis and determination of fatty acid composition of experimental concentrate diets was performed by Otto, et al. [23]

### Cooking protocol

Samples designated for cooking were removed from −20 °C storage and placed in a domestic refrigerator at 4 °C overnight to defrost. Prior to cooking, each sample was denuded of subcutaneous fat with any visible overlaying fat removed. Samples were cooked using conductive dry-heat on a flat surface hotplate using a Barbeques Galore G Series 4 burner (input: 20 MJ/h per burner) grilling unit with the cooking temperature set to High. No oil or other additives were used, thus negating their potential influence on final nutritional parameters. Samples were cooked until attainment of an internal temperature of 70 °C degrees, measured using a digital hand-held insta-read food thermometer with the thermocouple inserted into the approximated geometric centre of the LTL muscle. After cooking, each meat sample was rested for approximately three minutes, then sliced from the bone and cut into relatively equally sized portions of ~1 cm^3^. Cooked sub-sample portions (10-15 g) were stored at −20 °C until required for laboratory analysis.

### Analysis of moisture, intramuscular fat and fatty acids

Analysis of the moisture content of LTL muscle from each raw and cooked loin chop was determined as per ISO Reference Method 4442 [31], being dried until constant mass was achieved by means of an air convection oven set to a temperature of 105 °C. Moisture content was expressed as a percentage of the difference of meat mass pre- and post-drying.

Intramuscular fat (IMF) extraction and analysis of fatty acids was performed following the stepwise laboratory lipid analysis protocol described previously in the literature [32]. IMF from each raw and cooked LTL muscle was extracted overnight from a 1 g sub-sample of non-homogenised muscle using a modified Bligh and Dyer protocol [33], extracted using CHl_3_:MeOH: H_2_O (1:2:0.8 *v*/v), with phase separation using CHCl_3_:Saline Milli-Q H_2_O (1:1 *v*/v), and rotary evaporation. This protocol was used to determine IMF content expressed as percentages of the extracted IMF from the LTL muscle. Fatty acid methyl esters (FAME) of the extracted IMF were prepared from a trans-methylated aliquot volume of the total IMF, extracted three times using hexane/dichloromethane at a ratio of 4:1. Analysis was performed via capillary gas chromatography (GC) on an Agilent Technologies 7890B GC equipped with a 7683B series auto sampler and flame ionization detector, fitted with a non-polar Equity™-1 fused silica capillary column (15 m × 0.1 mmi.d., 0.1 μmfilm thickness), an FID, and split/splitless injector was used. Helium acted as the carrier gas. Peaks were quantified by ChemStation software (Agilent Technologies). Additional analysis was performed to confirm identifications of fatty acid peaks by GC–mass spectrometric (GC–MS) using a Finnigan Thermoquest GCQ GC-MS fitted with an on-column injector and using Thermoquest Xcalibur software (Austin, TX, USA). Fatty acids derived from the extracted lipid from each sample were methylated and analysed in duplicate and the data was averaged prior to statistical analysis. Quantitative analysis (mg/100 g) of fatty acid content was calculated from %FA data obtained from fatty acid area output, using a lipid conversion factor (LCF) of 0.916 [34] as referenced by Greenfield and Southgate [35] using the equation as presented by Clayton [36]: mg/100 g = (Total lipid)*(LCF [0.916])*([%FA]/100)*1000. Atherogenic (IA) and thrombogenic (IT) indices were calculated as per Ulbricht and Southgate [37]: IA = [(4*14:0) +16:0] / [(∑PUFA) + (∑MUFA)]; IT = [14:0 + 16:0 + 18:0]/[(0.5*∑MUFA) + (0.5*∑ ω6) + (3*∑ ω3) + (ω3/ω6)]. The Indices of hypocholesterolemic to hypercholesterolemic fatty acids (h/H) was calculated according to Bessa, et al. [38]: h/H = (18:1ω9c + 18:2ω6 + 20:4ω6 + 18:3ω3 + 20:5ω3 + 22:5ω3 + 22:6ω3)/(14:0 + 16:0). Percentage of apparent nutrient retention values (ARV%) were calculated as per Murphy, et al. [39]: ARV% = [nutrient content per g of cooked food (dry basis)]/[nutrient content per g of raw food (dry basis)] * 100.

### Statistical analysis

Statistical testing was performed using Statistical Analysis System (SAS) software version 9.2 (SAS Institute, Cary, NJ, USA) [40]. Summary statistics were first computed and included means and standard errors, appraisal for data entry errors and obvious outliers. ANOVA mixed linear model procedures were employed to test for fixed effects and two-way interactions. Diet, breed of sire and gender were fitted as main effects in the analytical model, with meat preparation as a covariate and fatty acids and retention values as dependent variables. Where significant (*P* < 0.05), pairwise comparisons using Tukey tests were implemented to establish differences between means.

## Results

The primary focus of this study was to determine the effects of dietary degummed crude canola oil supplementation and its interactions with sire breed and gender on selected nutrients (moisture and intramuscular fat content), fatty acid composition and content, and retention values in raw and cooked meats. Therefore, examination of the direct effect of cooking treatment on these parameters was not gauged.

### Proximate compositions and fatty acid profiles of experimental feeds

Proximate compositions and fatty acid profiles of the two primary concentrate rations (with and without the addition of degummed crude canola oil) used during the experimental period are given in Table 1. The concentrate rations were formulated to a metabolisable energy (ME) value of 11.4-12.1 MJ/kg DM, 10.4-12.7% DM Crude protein (CP) and 2.1-6.2% DM ether extractable (EE) fat between the concentrates with and without degummed canola oil, respectively. The concentrate without added DCCO contained greater proportions of 18:2ω6 (6.9-17.7% of total fatty acids, TFA), 18:3ω3 (0.5-1.6%), ∑PUFA (12.6-35.0%), ∑ω3 (0.9-14.9%), and ∑ω6 (10.2-20.1%), whereas the DCCO added concentrate contained a greater proportion of ∑MUFA than the non-added oil concentrate, accounting for 48.7% and 23.3% of the TFA, respectively. Oleic acid (18:1ω9c) was the main MUFA constituting 41.9% of the TFA for the DCCO added concentrate.

### Degummed crude canola oil supplementation

Feeding regime effects on moisture, intramuscular fat content, and fatty acid properties are presented in Table 2. The results show there was no difference (*P* > 0.05) between feeding regimes for moisture or intramuscular fat content percentage, averaging 71.1 – 60.2% and 3.4 – 5.1% between diets for these nutrients within raw and cooked meats, respectively. In the raw state, lambs fed the DCCOH diet had significantly increased (*P* < 0.05) concentrations of EPA (21.0 mg/100 g muscle) compared to the DCCOC (17.9 mg/100 g) and DCCOM (16.1 mg/100 g) diets. The sum total of EPA + DHA was likewise highest for the DCCOH (28.2 mg/100 g), but only significantly (*P* < 0.05) compared to the DCCOM diet (20.0 mg/100 g). In the cooked state, ω3 LC-PUFA concentrations increased linearly with the level of dietary oil inclusion; however, unlike observed for the raw state, there was no statistical difference (*P* > 0.05) between feeding regimes. Excluding the sum total of EPA + DHA in raw meats, the results did not reflect any dietary treatment effect (*P* > 0.05) on the sum total of fatty acid groupings within raw or cooked states. Likewise, there was no dietary effect (*P* > 0.05) on P/S or ω6/ω3 ratios, or for calculations of thrombogenicity (IT) and the ratio of hypocholesterolemic/hypercholesterolemic (h/H). Within raw meats, there was no difference (*P* > 0.05) for calculated index of atherogenicity (IA) between dietary regimes; however, in the cooked product indices of IA significantly declined (*P* < 0.05) with the addition of DCCO to the diet compared to the control diet.

**Table 2:** Effect of degummed crude canola oil feeding regime on raw and cooked lamb meat quality (LSM ± SEM)

	Raw meats			Cooked meats		
Fatty acids^A^	DCCOC	DCCOM	DCCOH	DCCOC	DCCOM	DCCOH
MC (%)	70.7 ± 0.6	72.0 ± 0.6	72.3 ± 0.5	61.6 ± 1.0	59.7 ± 1.6	59.4 ± 1.7
IMF (%)	3.5 ± 0.4	3.7 ± 0.6	2.9 ± 0.3	5.5 ± 0.6	5.0 ± 0.6	4.7 ± 0.6
∑FA	3203 ± 379.3	3421 ± 520.3	2628 ± 273.6	5030 ± 591.9	4565 ± 527.7	4264 ± 554.7
14:0	55.4 ± 10.7	55.7 ± 12.4	37.2 ± 5.0	113.5 ± 21.9	59.6 ± 11.1	59.7 ± 7.8
16:0	722.6 ± 88.5	769.4 ± 129.2	549.1 ± 57.8	1139.0 ± 129.0	956.1 ± 116.2	890.7 ± 119.6
18.0	537.2 ± 81.1	606.6 ± 98.5	434.7 ± 53.9	834.5 ± 109.5	833.6 ± 11.9	726.7 ± 102.2
16:1ω7c	41.0 ± 5.4	39.6 ± 7.5	26.9 ± 2.9	71.6 ± 12.4	49.2 ± 6.8	45.5 ± 7.0
18:1ω9c	1188 ± 179.2	1258 ± 205.3	928.5 ± 106.9	1880 ± 240.8	1693 ± 221.7	1561 ± 210.6
18:1ω7c	58.9 ± 5.4	64.3 ± 8.5	59.4 ± 4.9	83.5 ± 8.8	86.5 ± 9.6	87.9 ± 13.3
18:1ω7t	50.1 ± 8.8	57.9 ± 12.8	58.5 ± 7.5	103.8 ± 26.0	88.1 ± 14.5	108.6 ± 16.9
18:2ω6 (LA)	148.1 ± 11.2	182.2 ± 22.1	165.4 ± 16.8	221.6 ± 17.9	256.2 ± 28.0	231.6 ± 28.3
18:3ω3 (ALA)	27.6 ± 4.2	32.8 ± 4.7	33.1 ± 4.1	49.0 ± 9.0	47.7 ± 5.5	48.7 ± 7.6
20:4ω6 (ARA)	43.8 ± 4.1	43.0 ± 4.9	46.8 ± 2.4	59.2 ± 8.8	66.5 ± 7.9	62.7 ± 7.7
20:5ω3 (EPA)	17.9 ± 1.6^b^	16.1 ± 1.4^b^	21.0 ± 2.0^a^	21.4 ± 2.2	24.2 ± 1.8	28.1 ± 4.0
22:6ω3 (DHA)	6.1 ± 1.3	3.9 ± 0.5	7.2 ± 0.7	7.2 ± 0.8	7.6 ± 0.8	10.0 ± 1.9
22:5ω3 (DPA)	14.2 ± 1.5	14.2 ± 1.2	16.3 ± 0.8	20.7 ± 2.2	23.5 ± 2.0	23.2 ± 2.9
∑SFA	1453 ± 170.6	1562 ± 254.9	1137 ± 130.2	2292 ± 265.8	2031 ± 252.2	1853 ± 242.9
∑MUFA	1448 ± 202.1	1527 ± 246.9	1164 ± 128.4	2307 ± 309.8	2055 ± 267.1	1951 ± 259.1
∑PUFA	301.8 ± 19.2	331.2 ± 34.5	326.9 ± 25.5	430.5 ± 33.3	479.3 ± 43.7	460.8 ± 54.9
∑CLA	12.8 ± 2.1	12.5 ± 1.9	9.7 ± 1.0	17.6 ± 1.4	15.9 ± 2.1	18.1 ± 3.0
∑ω6	208.2 ± 14.1	238.9 ± 27.1	226.0 ± 19.6	298.6 ± 26.5	343.0 ± 36.5	314.4 ± 36.9
∑ω3	70.0 ± 6.0	69.7 ± 6.4	80.3 ± 7.1	101.9 ± 8.7	106.3 ± 6.2	113.9 ± 15.4
EPA + DHA	24.0 ± 2.9^ab^	20.0 ± 1.6^b^	28.2 ± 2.7^a^	28.6 ± 2.7	31.7 ± 2.4	38.1 ± 5.9
P/S	0.2 ± 0.0	0.2 ± 0.0	0.3 ± 0.0	0.2 ± 0.0	0.3 ± 0.0	0.3 ± 0.0
6/3	3.1 ± 0.2	3.5 ± 0.3	2.9 ± 0.2	3.0 ± 0.3	3.2 ± 0.2	2.8 ± 0.2
IA	0.5 ± 0.0	0.5 ± 0.0	0.5 ± 0.0	0.6 ± 0.0	0.5 ± 0.0	0.5 ± 0.0
IT	1.2 ± 0.1	1.3 ± 0.1	1.1 ± 0.0	1.3 ± 0.0	1.2 ± 0.0	1.1 ± 0.0
h/H	1.9 ± 0.1	2.0 ± 0.1	2.1 ± 0.0	1.8 ± 0.1	2.1 ± 0.1	2.1 ± 0.1

^A^MC (%) = Moisture content percentage. IMF (%) = Intramuscular fat content percentage. ∑FA is the sum total of SFA, MUFA, PUFA; ∑SFA is the sum of 14:0, 15:0, 16:0, 17:0, 18:0, i15:0, a15:0, i16:0, 16:0FALD, i17:0, i18:0, 18:0FALD, 20:0, 22:0, 23:0, 24:0; ∑MUFA is the sum of 16:1ω7c, 18:1ω9c, 18:1ω7c, 18:1ω7t, 14:1, 16:1ω9c, 16:1ω7t, 16:1ω5c, 16:1ω13t, 17:1ω8c + a17:0, 17:1, 18:1a, 18:1b, 18:1c, 19:1a, 19:1b, 18:1FALD, 20:1ω11c, 20:1ω9c, 20:1ω7c, 20:1ω5c, 22:1ω11c, 22:1ω9c, 24:1ω9c; ∑PUFA is the sum of 18:2ω6, 18:3ω3, 20:4ω6, 20:5ω3, 22:6ω3, 22:5ω3, 18:3ω6, 18:4ω3, 18:2ω6CLAa, 18:2ω6CLAb, 18:2ω6CLAc, 20:3, 20:3ω6, 20:4ω3, 20:2ω6, 22:5ω6, 22:4ω6; ∑CLA is the sum total 18:2CLAa, 18:2CLAb, 18:2CLAc; ∑ω-6 is the sum of 18:2ω6, 20:4ω6, 18:3ω6, 20:3ω6, 20:2ω6, 22:5ω6, 22:4ω6; ∑ω-3 is the sum of 18:3ω3, 20:5ω3, 22:6ω3, 22:5ω3, 18:4ω3, 20:4ω3; ∑EPA + DHA is the sum of 22:6ω3 and 20:5ω3; P/S is ∑PUFA/∑SFA; 6/3 is ∑ω6/∑ω3; IA = Atherogenic index; IT = Thrombogenic index; h/H = hypocholesterolemic to hypercholesterolemic index. DCCOC = 0 mL/kg DM DCCO. DCCOM = 25 mL/kg DM DCCO. DCCOH = 50 mL/kg DCCO. Fatty acids are presented on an mg/100 g muscle tissue basis. Values are least square means and standard error of the means for 6 DCCOC, 7 DCCOM, and 7 DDCOH supplemented lambs within raw and cooked analysis. Row means within raw and cooked meats showing differing superscript letters significantly differ (*P* < 0.05)

### Sire breed

Sire breed fixed effects are presented in Table 3. Results show that breed of sire had no effect (*P* > 0.05) on either moisture or IMF content within raw or cooked meats. In the raw state, EPA and the sum total of EPA + DHA were significantly (*P* < 0.05) influenced by sire breed genetics, with lambs from Dorset sired progeny displaying a greater propensity for ω3 LC-PUFA deposition than the other breeds of sire studied. Corresponding to the raw state, ω3 LC-PUFA concentration in cooked meat was greater under the influence of Dorset sire genetics; however, the observed significant differences between sires for ω3 LC-PUFA content in raw meats were not statistically evident (*P* > 0.05). All summations, ratios and indices, except the sum total of EPA + DHA in raw meat, were shown to be not influenced (*P* > 0.05) by breed of sire effect for meats in both the raw and cooked states.

**Table 3:** Breed of sire influence on raw and cooked lamb meat quality (LSM ± SEM)

	Raw meats			Cooked meats		
Fatty acids^A^	Dorset	White Suffolk	Merino	Dorset	White Suffolk	Merino
MC (%)	71.4 ± 0.5	72.4 ± 0.4	70.9 ± 1.1	60.8 ± 1.2	60.3 ± 1.4	59.0 ± 2.0
IMF (%)	3.5 ± 0.3	3.1 ± 0.5	3.9 ± 0.7	5.9 ± 0.7	4.3 ± 0.4	5.0 ± 0.6
∑FA	3198 ± 234	2797 ± 437	3542 ± 676	5375 ± 615	3976 ± 350	4573 ± 514
14:0	53.6 ± 8.7	42.1 ± 10.0	58.4 ± 14.1	98.7 ± 22.1	57.4 ± 8.6	76.7 ± 13.7
16:0	701.4 ± 51.2	606.4 ± 101.8	813.6 ± 181.9	1149 ± 139	865. ± 82.4	975.9 ± 132
18.0	570.7 ± 57.6	459.3 ± 78.3	602.2 ± 139.4	961.8 ± 109	654.0 ± 64.1	809.3 ± 116
16:1ω7c	35.1 ± 3.9	32.8 ± 6.4	44.2 ± 9.0	65.1 ± 12.6	47.8 ± 6.0	52.2 ± 7.1
18:1ω9c	1143 ± 98.4	1009 ± 174	1357 ± 293	1978 ± 260	1491 ± 143	1681 ± 219
18:1ω7c	58.4 ± 4.6	62.6 ± 7.2	62.6 ± 8.6	90.6 ± 10.9	85.4 ± 9.9	79.0 ± 7.4
18:1ω7t	59.3 ± 7.7	54.2 ± 11.1	52.0 ± 11.7	121.1 ± 22.0	83.5 ± 12.4	93.6 ± 20.2
18:2ω6 (LA)	166.1 ± 8.1	162.0 ± 22.0	174.4 ± 23.2	260.6 ± 19.1	213.7 ± 27.7	244.7 ± 18.3
18:3ω3 (ALA)	36.2 ± 3.2	27.6 ± 4.1	29.7 ± 6.0	61.4 ± 7.5	39.3 ± 3.5	44.1 ± 6.2
20:4ω6 (ARA)	45.5 ± 3.5	44.5 ± 3.8	42.5 ± 5.5	64.5 ± 7.2	62.2 ± 9.1	62.0 ± 3.2
20:5ω3 (EPA)	21.0 ± 1.7^a^	16.6 ± 1.4^b^	16.6 ± 2.0^b^	26.6 ± 3.1	23.7 ± 2.6	22.7 ± 2.5
22:6ω3 (DHA)	6.7 ± 0.8	5.6 ± 0.9	3.8 ± 1.4	9.4 ± 1.4	8.1 ± 1.1	6.3 ± 0.8
22:5ω3 (DPA)	15.5 ± 1.0	15.6 ± 1.0	12.3 ± 1.6	23.5 ± 1.9	22.7 ± 2.7	20.6 ± 1.5
∑SFA	1464 ± 110.0	1228 ± 206.0	1600 ± 344.3	2432 ± 275	1727 ± 156	2062 ± 250
∑MUFA	1405 ± 118	1254 ± 209	1624 ± 336	2438 ± 326	1828 ± 176	2060 ± 243
∑PUFA	329.8 ± 16.7	314.0 ± 31.3	317.5 ± 36.8	504.4 ± 31.3	420.7 ± 49.0	451.4 ± 28.1
∑CLA	10.8 ± 1.1	12.5 ± 2.0	11.8 ± 2.1	19.6 ± 2.2	14.1 ± 1.4	18.7 ± 2.9
∑ω6	225.7 ± 11.4	221.8 ± 25.8	230.8 ± 26.7	344.6 ± 24.4	295.8 ± 39.1	325.1 ± 20.2
∑ω3	82.2 ± 5.8	68.8 ± 5.3	66.2 ± 8.2	124.9 ± 7.9	97.4 ± 9.2	96.1 ± 7.2
EPA + DHA	27.7 ± 2.4^a^	22.2 ± 2.1^b^	20.4 ± 3.3^b^	36.0 ± 4.5	31.8 ± 3.6	29.1 ± 2.7
P/S	0.2 ± 0.0	0.3 ± 0.0	0.2 ± 0.1	0.2 ± 0.0	0.3 ± 0.0	0.2 ± 0.0
ω6/ω3	2.8 ± 0.1	3.3 ± 0.3	3.6 ± 0.1	2.8 ± 0.2	3.0 ± 0.2	3.4 ± 0.1
IA	0.5 ± 0.0	0.5 ± 0.0	0.5 ± 0.0	0.5 ± 0.0	0.5 ± 0.0	0.5 ± 0.0
IT	1.2 ± 0.1	1.1 ± 0.0	1.3 ± 0.1	1.2 ± 0.0	1.2 ± 0.0	1.2 ± 0.0
h/H	1.9 ± 0.1	2.0 ± 0.1	1.9 ± 0.1	2.0 ± 0.1	2.1 ± 0.1	2.0 ± 0.1

^**A**^Fatty acids are as defined in Table 2. Fatty acids are presented on an mg/100 g muscle tissue basis. Values are least square means and standard error of the means for 8 Dorset, 8 White Suffolk, and 4 Merino sired lambs within raw and cooked analysis. Row means within raw and cooked meats showing differing superscript letters significantly differ (*P* < 0.05)

### Gender

The results, as presented in Table 4, report no difference (*P* > 0.05) between ewe and wether lambs for moisture content, IMF content, concentrations of individual fatty acids, their partial sums, ratios and indices within either raw or cooked meats.

**Table 4:** Gender influence on raw and cooked lamb meat quality (LSM ± SEM)

	Raw meat		Cooked meat	
Fatty acids^A^	Ewe	Wether	Ewe	Wether
MC (%)	72.6 ± 0.6	71.2 ± 0.4	61.4 ± 1.1	59.5 ± 1.1
IMF (%)	3.2 ± 0.5	3.5 ± 0.3	5.8 ± 0.6	4.6 ± 0.4
∑FA	2963 ± 475.2	3182 ± 280.1	5292 ± 548.9	4223 ± 342.9
14:0	50.2 ± 13.7	49.5 ± 5.6	90.3 ± 24.4	68.7 ± 6.9
16:0	637.9 ± 112.4	712.6 ± 70.2	1123 ± 135.5	917.4 ± 74.7
18.0	481.2 ± 86.4	559.2 ± 58.2	908.7 ± 102.4	736.8 ± 71.5
16:1ω7c	35.3 ± 7.1	36.4 ± 4.0	65.9 ± 12.6	48.8 ± 4.4
18:1ω9c	1055 ± 186.0	1177 ± 121.1	1990 ± 222.1	1547 ± 137.7
18:1ω7c	61.5 ± 7.6	60.8 ± 4.2	103.7 ± 9.4	75.7 ± 5.8
18:1ω7t	59.2 ± 12.6	53.5 ± 5.8	128.3 ± 20.2	82.7 ± 9.9
18:2ω6 (LA)	174.7 ± 22.9	161.1 ± 10.2	265.7 ± 27.6	221.1 ± 15.0
18:3ω3 (ALA)	31.9 ± 4.5	30.8 ± 3.0	57.5 ± 7.1	43.2 ± 4.3
20:4ω6 (ARA)	48.4 ± 3.0	42.2 ± 3.0	66.3 ± 9.8	61.1 ± 4.6
20:5ω3 (EPA)	19.2 ± 1.5	17.6 ± 1.4	24.3 ± 2.5	24.7 ± 2.2
22:6ω3 (DHA)	6.1 ± 1.0	5.4 ± 0.7	7.9 ± 1.1	8.4 ± 1.0
22:5ω3 (DPA)	16.7 ± 0.9	13.8 ± 0.8	23.6 ± 2.7	21.9 ± 1.5
∑SFA	1307 ± 225.5	1444 ± 137.9	2324 ± 272.4	1902 ± 156.7
∑MUFA	1314 ± 224.8	1430 ± 139.1	2464 ± 280.6	1890 ± 163.6
∑PUFA	341.9 ± 29.6	308.1 ± 17.8	503.9 ± 43.6	431.3 ± 28.7
∑CLA	13.4 ± 2.2	10.7 ± 0.9	19.3 ± 1.8	15.8 ± 1.6
∑ω6	239.4 ± 25.7	216.8 ± 12.8	352.3 ± 38.2	301.1 ± 19.8
∑ω3	77.5 ± 5.2	70.7 ± 5.1	117.5 ± 6.4	101.3 ± 8.1
EPA + DHA	25.3 ± 2.3	23.0 ± 2.0	32.2 ± 3.5	33.1 ± 3.1
P/S	0.3 ± 0.0	0.2 ± 0.0	0.2 ± 0.0	0.2 ± 0.0
ω6/ω3	3.2 ± 0.3	3.2 ± 0.1	3.0 ± 0.3	3.0 ± 0.1
IA	0.5 ± 0.0	0.5 ± 0.0	0.5 ± 0.0	0.5 ± 0.0
IT	1.1 ± 0.0	1.2 ± 0.0	1.2 ± 0.1	1.2 ± 0.0
h/H	2.0 ± 0.1	1.9 ± 0.1	2.1 ± 0.1	2.0 ± 0.0

^**A**^Fatty acids are as defined in Table 2. Fatty acids are presented on an mg/100 g muscle tissue basis. Values are least square means and standard error of the means for 8 ewe and 12 wether lambs within raw and cooked analysis. Row means within raw and cooked meats showing differing superscript letters significantly differ (*P* < 0.05)

### Interactions

**Fig. 1:**
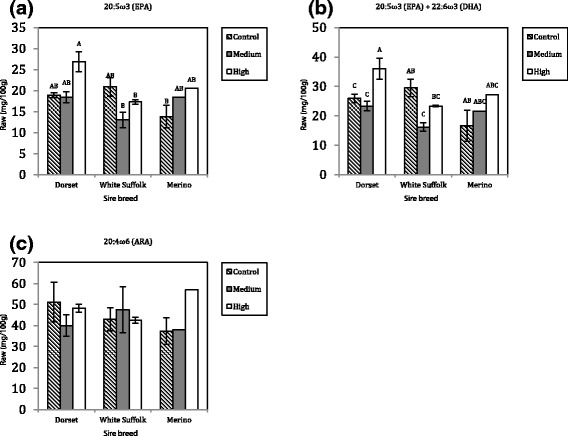
Feeding regime by sire breeds interactions in raw meat. **a** EPA; **b**; EPA + DHA; **c** ARA

In the raw meat, significant (*P* < 0.05) sire breed by diet interactions were evident for EPA, EPA + DHA, and 20:4ω6 (ARA) (Fig. 1). In terms of overall effect, both muscle EPA and EPA + DHA concentration showed similar patterns of response whereby the concentration of these fatty acids in the muscle of Dorset and Merino sired lambs tended to increase with the volume of DCCO added in the diet, whereas for White Suffolk sired lambs the concentrations of these fatty acids tended to decline. In terms of ARA concentration, pure Merino demonstrated a greater concentration of this fatty acid within the muscle as a response to the DCCOH level of supplementation compared to the other diets, whereas for Dorset and White Suffolk the greatest response was from the DCCOM level of supplementation which shown to increase and decrease in concentration for these sire breeds, respectively.

**Fig. 2:**
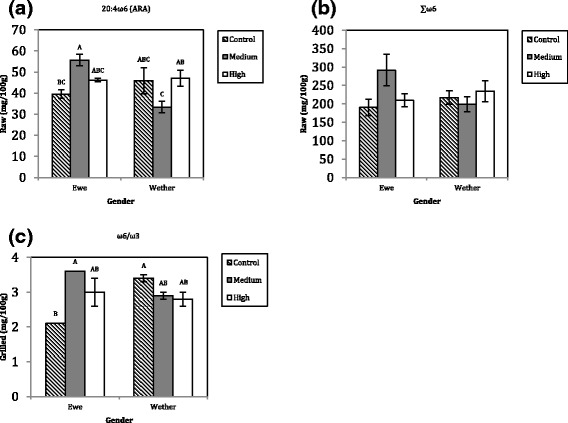
Feeding regime and gender interactions. **a** ARA; **b** ∑ω6 raw; **c** ω6/ω3 cooked meats

Additionally in the raw state, a gender by diet interaction effect (*P* < 0.05) was identified for ARA and ∑ω6 (Fig. 2). These results were mainly derived from differences between ewes and wethers under the Medium (DCCOM) level of supplementation. In the cooked state, a significant (*P* < 0.05) ω6/ω3 difference occurred between gender and dietary supplementation (Fig. 2), with the major influence between ewe and wether lambs under the control (DCCOC) diet, and between the control diet and medium level (DCCOM) of supplementation within ewes. All other interactions within raw or cooked meats were shown not to be significant (*P* > 0.05); therefore this data has been omitted from the manuscript.

### Retention of nutrients

Coefficients of apparent nutrient retention are presented in Table 5. Feeding regime or sire breed was shown not to be a factor (*P* > 0.05) in the ability of meats to retain nutrients (ARV %) as a function of the cooking process. In terms of gender, meat from ewe lambs demonstrated significantly (*P* < 0.05) superior ARV% than wethers for IMF/sum total of fatty acids, and the sum totals of SFA and MUFA, as well as for the major individual fatty acids comprising the overall fatty acid profile (18:1ω9c, 16:0, 18:0). In effect, the ARV% of these values exceeded 100% for ewe lambs, whereas for wethers these were consistently less than 100%.

**Table 5:** Apparent retention values of fatty acids based on feeding regime, sire breed and gender

	Feeding regime			Sire breed			Gender	
Fatty acids^A^	DCCOC	DCCOM	DCCOH	Dorset	White Suffolk	Merino	Ewe	Wether
Moisture (%)	67.7 ± 3.3	61.2 ± 3.0	52.2 ± 4.5	62.6 ± 3.6	58.9 ± 3.9	60.7 ± 6.9	62.1 ± 3.9	59.9 ± 3.2
IMF (%)	126.3 ± 18.2	107.2 ± 19.9	103.9 ± 8.4	127.2 ± 21.3	107.1 ± 11.9	96.2 ± 7.3	141.1 ± 20.6^a^	95.3 ± 5.0^b^
∑fatty acids	126.3 ± 18.2	107.2 ± 19.9	103.9 ± 8.4	127.2 ± 21.3	107.1 ± 11.9	96.2 ± 7.3	141.1 ± 20.6^a^	95.3 ± 5.0^b^
14:0	163.5 ± 15.2	105.8 ± 36.8	105.3 ± 6.7	144.6 ± 33.2	112.4 ± 17.7	110.4 ± 29.3	155.2 ± 35.8	105.6 ± 10.4
16:0	128.0 ± 18.4	103.0 ± 22.0	104.0 ± 7.6	124.8 ± 21.9	109.5 ± 13.9	91.0 ± 8.6	141.2 ± 22.5^a^	93.7 ± 5.1^b^
18.0	128.1 ± 21.6	114.8 ± 26.3	107.7 ± 11.9	134.3 ± 27.8	109.3 ± 14.6	100.9 ± 7.1	153.7 ± 25.7^a^	95.2 ± 6.6^b^
16:1ω7c	139.5 ± 20.8	103.7 ± 22.0	109.0 ± 11.3	136.3 ± 23.1	112.6 ± 14.2	90.5 ± 14.3	146.5 ± 24.4	99.3 ± 7.2
18:1ω9c	134.8 ± 26.0	107.9 ± 20.7	108.0 ± 8.1	132.6 ± 25.1	113.8 ± 14.7	93.3 ± 6.6	152.2 ± 24.4^a^	95.6 ± 5.5^b^
18:1ω7c	112.6 ± 16.2	103.0 ± 14.7	92.8 ± 8.6	115.7 ± 17.9	96.9 ± 8.9	92.0 ± 6.7	127.9 ± 16.4^a^	88.2 ± 3.9^b^
18:1ω7t	166.5 ± 36.1	135.6 ± 37.3	118.2 ± 13.7	167.8 ± 44.6	117.9 ± 15.6	134.8 ± 17.6	187.6 ± 41.1^a^	112.0 ± 10.3^b^
18:2ω6 (LA)	115.6 ± 8.6	107.2 ± 12.5	91.6 ± 11.5	113.8 ± 8.0	96.6 ± 11.3	106.3 ± 17.3	114.8 ± 11.9	99.2 ± 7.5
18:3ω3 (ALA)	140.1 ± 19.1	115.6 ± 18.2	93.9 ± 9.2	127.9 ± 20.1	107.9 ± 14.4	113.6 ± 18.0	140.7 ± 20.7	102.4 ± 8.5
20:4ω6 (ARA)	104.8 ± 15.7	117.6 ± 15.5	87.4 ± 11.3	101.2 ± 7.3	101.0 ± 15.8	115.0 ± 24.8	95.0 ± 9.4	109.3 ± 12.2
20:5ω3 (EPA)	92.9 ± 7.4	114.4 ± 15.5	85.9 ± 9.2	91.3 ± 7.5	103.8 ± 14.8	100.8 ± 12.7	90.2 ± 7.7	103.5 ± 10.3
22:6ω3 (DHA)	254.8 ± 169.0	169.0 ± 44.7	90.7 ± 14.2	104.3 ± 13.5	137.5 ± 43.2	356.4 ± 247.9	129.6 ± 49.3	195.7 ± 82.8
22:5ω3 (DPA)	120.0 ± 21.7	126.5 ± 16.7	92.3 ± 11.3	109.8 ± 8.5	107.0 ± 17.0	133.6 ± 32.3	101.1 ± 12.0	121.0 ± 14.0
∑SFA	126.2 ± 16.8	107.5 ± 22.8	104.8 ± 8.5	127.4 ± 22.8	107.3 ± 12.6	97.3 ± 8.4	142.2 ± 22.3^a^	95.3 ± 5.3^b^
∑MUFA	132.6 ± 24.2	108.1 ± 20.9	107.2 ± 8.0	132.6 ± 25.0	110.8 ± 13.1	95.3 ± 7.0	149.9 ± 23.7^a^	95.5 ± 5.2^b^
∑PUFA	110.3 ± 9.4	109.6 ± 12.1	91.4 ± 10.7	110.5 ± 5.3	96.9 ± 12.1	107.2 ± 17.3	108.3 ± 10.4	101.6 ± 8.2
∑CLA	119.1 ± 19.9	108.7 ± 28.2	119.0 ± 15.0	143.0 ± 25.7	88.8 ± 12.6	119.7 ± 20.1	125.2 ± 27.6	109.5 ± 12.2
∑ω6	110.4 ± 9.0	108.7 ± 12.1	90.8 ± 11.2	110.0 ± 5.6	96.5 ± 11.9	106.5 ± 17.8	108.2 ± 10.6	100.9 ± 8.2
∑ω3	113.2 ± 9.9	115.8 ± 12.8	91.1 ± 9.4	110.9 ± 6.2	103.1 ± 13.2	108.9 ± 16.2	111.3 ± 10.3	104.8 ± 8.8
EPA + DHA	96.8 ± 12.0	120.1 ± 15.7	87.1 ± 10.4	94.2 ± 8.8	105.8 ± 15.8	109.7 ± 18.0	92.8 ± 11.0	107.9 ± 10.9

^**A**^Fatty acids are as defined in Table 2. Retention values are least square means and standard error of the means for anatomically paired raw and cooked meats from the same animal based on 6 DCCOC, 7 DCCOM, 7 DCCOH, 8 Dorset, 8 White Suffolk, 4 Merino, 8 ewe and 12 wether lambs. Data means within the same row within fatty acids within production variable effect with different superscript letters significantly differ (*P* < 0.05)

## Discussion

### Degummed crude canola oil supplementation

In this study, both moisture and intramuscular fat content percentages of the LTL muscle were not dependent on the inclusion of oil in the diet regardless of cooking state. Moisture content observations are consistent with previous studies [38, 41, 42] reporting water-holding properties of small ruminant meats. In terms of IMF, the lack of difference between dietary groups herein is consistent with previous findings for raw longissimus muscle from lambs supplemented with canola seeds and meals [43, 44]. Increases in IMF content in cooked meats without significant effect amongst dietary treatments correspond with the non-significant reduction in meat moisture content post-cooking, and is a finding consistent with the inverse relationship between these two meat properties.

In the raw state, the concentrations (mg/100 g muscle) of ω3 LC-PUFA (EPA, 21 mg/100 g; DHA, 7.2 mg/100 g; DPA 16 mg/100 g) for the DCCOH diet were similar to those reported by Ponnampalam, et al. [44] for the *longissimus* muscle of Australian 2nd-cross ([Merino × Border Leicester] × Poll Dorset) wether lambs supplemented with canola meal (EPA, 15.2 mg/100 g; DHA, 5 mg/100 g; DPA, 17 mg/100 g) and protected canola seed (EPA, 12.8 mg/100 g; DHA, 4.8 mg/100 g; DPA, 15 mg/100 g), albeit with concentrations of EPA and DHA in the current study slightly higher. Unlike the findings herein, however, Ponnampalam, et al. [44] did not detect significant ω3 LC-PUFA concentration effects with the application of either forms of canola when comparing to a control diet of mixed lucerne and oaten chaff offered at alternative ratios. In this respect, it can be argued that despite these forms of canola providing relatively high amounts of α-linolenic acid (ALA, 18:3ω3; 1404-3459 mg/total ω3 intake/day for canola meal and protected seed, respectively), there was insufficient rumen by-pass from these feed sources to induce increases in the ω3 LC-PUFA content of muscle tissue. This observation has been reported by Karami, et al. [45], when they compared canola oil with palm oil supplementation for compositional changes in the *longissimus* muscle tissue of goats. Results herein suggest, however, that the higher volume of lipid associated with the DCCOH diet did not seem to impair rumen microbial activities, and that this dietary oil source was more efficient than the other diets (particularly compared to the DCCOM diet) in utilising the ALA conversion to and deposition of ω3 LC-PUFA into the muscle. Essentially, this indicates that the physiochemical characteristics of DCCOH and mode of application influenced the degree to which dietary oil escaped rumen biohydrogenation. Alternatively, the meat from DCCOH supplemented lambs may have contained a greater proportion of phospholipids as a percentage of the total lipids, a factor that increases the concentration of PUFA in meats [5, 12, 17].

The observed non-significant ω3 LC-PUFA results between feeding regimes in the cooked state suggests some meats within dietary treatments may have displayed a greater susceptibility to heat induced oxidative degradation of ω3 LC-PUFA than others. On assessment of the data, it appears this was likely due to the non-significant differences in ARV % for these fatty acid types, particularly with reference to the lower rates of retention of ω3 LC-PUFA under DCCOH supplementation compared to the other diets. In previous research, Badiani, et al. [25] and Maranesi, et al. [27] suggested that cooking processes can differ between meats due to variation in physical composition, namely shape, size and surface/volume ratio. These properties may have influenced the results herein as meats were not standardised prior to cooking, but rather were prepared at the time of carcass dissection, albeit with subcutaneous fat removed. In effect, individual variation of meat properties within dietary regimes may have been a driving factor for this observation.

### Sire breed

The lack of significant difference in moisture content between sire breeds herein is consistent with previous studies [46, 47] reporting between crossbred sheep within raw meats; however, there has been some discrepancy between studies in the water-holding capacity of meats as a function of sheep breed as affected by cooking processes when assessing shear force measurements [46, 48]. IMF content percentage levels between sires in the raw LTL ranging 3.1-3.9% within raw meats were comparative to the calculated 4% mean value reported amongst pure-bred and 1st cross for Australian first-cross prime lambs when examined at around the same age at slaughter, and likewise showing no significant effect on IMF proportions [49].

In the raw state, meats sourced from Dorset sired progeny displayed a greater propensity for ω3 LC-PUFA deposition than those of other sires types. Australian sheep industry CRC research reported by Ponnampalam, et al. [20] has identified that LC-PUFA content, namely EPA + DHA, increases linearly with the degree of influence of Merino input. Hence, it was anticipated that purebred Merino in the current study would have a tendency for increased ω3 LC-PUFA deposition compared to the other sire breeds. This was not the case. Such observations are explained by Pannier, et al. [18] and Ponnampalam, et al. [19] stating that differences between sire breeds, whilst significant, are comparatively minor, and are mainly dependent upon site of production, kill date, and individual animal variation, rather than sire breed per se. In the cooked state, differences in ω3 LC-PUFA concentrations between sires were not statistically evident (*P* > 0.05); a result which may have been influenced by physical characteristic differences between meats.

### Gender

Results between genders within raw meats are consistent with other reports of negligible gender effect on moisture properties [50–52] or IMF [50, 53, 54] percentages of lamb meat. In terms of ω3 LC-PUFA content, the findings herein contrast to those previously reported by Ponnampalam, et al. [19] reporting that although minor in nature, differences occur between sheep genders under Australian production systems for ω3 LC- PUFA deposition for the *longissimus* (on a fresh meat basis), with content in females superior to that of males. However, ω3 LC-PUFA concentration results within raw meats are in agreement with recent findings by Malau-Aduli, et al. [32] for these same lamb types at a similar age. Malau-Aduli, et al. [32] attributed these findings to a castration effect of the male lamb genotype which consequently negated any predetermined sex related hormonal differences to those of females.

### Interactions

Comparing with the published literature, the long-chain omega-3 interaction results between sire breed and feeding regime for the uncooked meats align with those of Demirel, et al. [21] who reported results between meat and milk producing sheep breeds under pasture and concentrate management regimes. Previously, De Smet, et al. [55] evaluated that diet plays a greater role on meat fatty acid composition than sheep breed, a finding that is in agreement with our results. From an Australian production viewpoint, the interaction results within raw meats observed herein contrast with those of Ponnampalam, et al. [20] reporting that when slaughtered at the same age, Australian cross bred prime lambs benefit from nutritional intervention to attain comparable ω3 LC-PUFA content to that contained in meat from purebred Merino.

Throughout the literature, competition in the Δ^6^-, Δ^5^-desaturase and elongation pathways between 18:2ω6 and 18:3ω3 for conversion to their respective long-chain 20:4ω6 and 20:5ω3/22:5ω3 derivatives are reported [56]. Current findings for conversion through in these pathways dictate that an excess in the conversion of a precursor fatty acid to its long-chain derivatives competes with and thereby limits conversion along the other pathway. From this, the interaction results between breed of sire and diet, showing comparative significance for EPA and EPA + DHA compared to ARA, advocates competition between 18:2ω6 and 18:3ω3 desaturase and elongation activities amongst sire breeds based on nutritional regime. Of note is the interaction result of purebred Merino fed the DCCOH level of supplementation demonstrating an increased ARA content in the raw muscle tissue that may in part, explain the differences in ω3 LC-PUFA content between sire breeds. As with sire breed by dietary interactions, results between gender and feeding regime within raw meats demonstrate nutritional regulation based on genetic predispositions amongst sheep types, which may again be due to competition between the ω6 and ω3 pathways as defined above.

### Nutritional contribution of cooked lamb meat to the human diet

In a bid to provide information regarding the contribution of lamb meat to supply ω3 LC-PUFA to the human diet, some reports define minimum EPA + DHA concentrations for raw meats at 24 mg/100 g [18, 57] or 30 mg/135 g [58], corresponding to Australian defined ‘source’ level ω3 LC-PUFA at 30 mg/100 g of cooked lamb meat [59]. However, for purposes of defining the nutritional contributions of the experimental meats to consumers, fatty acid results herein were appraised with 100 g of the cooked lean constituting a single serve portion. Between dietary oil treatments, both the DCCOM (32 mg/100 g EPA + DHA) and DCCOH (38 mg/100 g EPA + DHA) supplementation achieved source level LC-PUFA content per single serving of cooked lean meat product, whereas the DCCOC diet (29 mg/100 g) narrowly failed to achieve this status. Between sire breeds, the method of cooking used identified meats from Dorset (36 mg/100 g) and White Suffolk (32 mg/100 g) sired lambs as attaining source level content, whereas purebred Merino (29 mg/100 g) did not. Both ewe (32 mg/100 g) and wether (33 mg/100 g) lambs achieved source level health claimable ω3 LC-PUFA content for the cooked meats. When the value of docosapentaenoic acid (22:5ω3; DPA) was added to all effects, the ω3 LC-PUFA (EPA + DHA + DPA) content of the cooked meats contributed 55 mg to the 160-90 mg recommended daily adequate intake for adult men and women, respectively [60]. These findings agree with those of Ponnampalam, et al. [20] indicating that lamb meats have the capacity to contribute substantially to an adequate supply of health beneficial ω3 LC-PUFA to the diet where fish sources are not available or readily consumed. However, as identified herein, ω3 LC-PUFA contributions to the diet may depend upon on-farm production inputs. The application of the cooking method employed may likewise be a factor.

In terms of nutritional ratios and indices, all cooked meats, irrespective of effects analysed, demonstrated P/S ratios that were lower than the recommended minimum value of 0.45 ideal for the human diet [61]. The ω6/ω3 values for all cooked meats were lower than the maximum acceptable value of 4.0 [61]. Atherogenicity (IA) and thrombogenicity (IT) are indices based on assessing atheroma and thrombus formation [37], with the premise that lower values indicate a healthier product in terms of reducing the incidence of coronary heart disease. Within the final cooked product, indices of IA indicated that meats from both DCCOM and DCCOH supplemented lambs were more beneficial in reducing the risk of atheroma associated with coronary heart disease compared to meats from the DCCO diet. Measurement of IT regardless of the assessed effects were relative to the benchmarked value of 1.33 originally presented by Ulbricht and Southgate [37] for the lean component of lamb. h/H is a nutritional quality index measuring the cholesterol contribution of food types, with higher values indicating a healthier product. In our study h/H values ranging from 1.8-2.1 across all effects are in accordance with the nutritional mean of 2.11 for grilled lamb meats as presented by Campo, et al. [62].

### Retention of nutrients

Assessment of retention enables evaluation of the correct loss/degradation or increase of meat nutrients as a direct function of the cooking process [63]. Findings in the current study showing no significant production variable effects on ω3 LC-PUFA retention indicate that these nutrients generally behaved in the same manner for the cooking mode employed herein - a finding that is supported by our statistical analysis of the data when examining the direct effect of cooking with these dependents (data not presented). Moreover, this finding is corroborated by Alfaia, et al. [63] and Knight, et al. [64] who observed no significant diet by cooking, or breed by cooking interaction effect for fatty acids when examining these parameters for beef and lamb, respectively. The observed significant ARV % results between genders indicated that the culinary practice employed herein imparted some differences on meat lipid properties; however, when the data was re-assessed as cooking by gender interaction effect (data not presented) there were no differences for these fatty acids. Subsequently, these results indicate that differences of nutrient retention as a function of cooking as indicated in the fatty acid profiles between genders was not sufficient to warrant numerical difference of fatty acid content in meats based on these sheep types.

## Conclusions

The results from this study showed that the inclusion of degummed crude canola oil at 50 mL/kg in the sheep finishing ration is capable of providing consumers with a nutritionally beneficial product, particularly with reference to the key ω3 LC-PUFA. Our results further demonstrated that sire breed plays an important role in regulating ω3 LC-PUFA deposition in lamb meat for enhancing human nutrition. There was no difference between genders in their capacity to provide these key nutrients. Interaction results demonstrated the significance of feeding regime and genetic predisposition of sheep types on the lamb fatty acid profiles. However, it should be noted that significant differences between production variable effects were only observed for meats in the raw state. The cooking treatment employed did not induce differences in ω3 LC-PUFA retention as a function of production effects. Taken together, these results present that combinations of dietary degummed crude canola oil, sheep genetics, and culinary preparation method can be used as effective management tools to deliver nutritionally improved ω3 LC-PUFA lamb meats to consumers. Hence, the overall findings herein support the presented hypotheses for this study.

## Abbreviations

ARV%: Apparent retention value percentage
DCCOH: Degummed crude canola oil added at a volume of 50 mL/kg to the concentrate
DCCO: Degummed crude canola oil
DCCOC: 0 mL/kg DM added degummed crude canola oil to the concentrate
DCCOM: 25 mL/kg DM of degummed crude canola oil to the concentrate
DHA: Docosahexaenoic acid 22:6ω3
DPA: Docosapentaenoic acid 22:5ω3)
EPA + DHA: Sum of eicosapentaenoic and docosahexaenoic fatty acids
EPA: Eicosapentaenoic acid 20:5ω3
IMF: Intramuscular fat
PUFA: Polyunsaturated fatty acids
ω3 LC-PUFA: Long-chain [≥C_20_] omega-3 polyunsaturated fatty acids
